# The Effect of Breastfeeding Practices of Undernourished Mothers in Rural Sierra Leone on Infant Growth and Mortality

**DOI:** 10.3390/children11020233

**Published:** 2024-02-10

**Authors:** Aminata Shamit Koroma, Kevin B. Stephenson, Per O. Iversen, Mark J. Manary, David Taylor Hendrixson

**Affiliations:** 1 Ministry of Health, Republic of Sierra Leone, Freetown 00232, Sierra Leone; shamitamin@gmail.com; 2 Department of Medicine, Washington University School of Medicine, St. Louis, MO 63110, USA; k.stephenson@wustl.edu; 3 Department of Nutrition, University of Oslo, 0317 Oslo, Norway; p.o.iversen@medisin.uio.no; 4 Department of Haematology, Oslo University Hospital, 0450 Oslo, Norway; 5 Division of Human Nutrition, Stellenbosch University, Tygerberg 7505, South Africa; 6 Department of Pediatrics, Washington University School of Medicine, St. Louis, MO 63108, USA; 7 Department of Pediatrics, University of Washington School of Medicine, Seattle, WA 98195, USA; dhendrix@uw.edu

**Keywords:** exclusive breastfeeding, infant growth, infant mortality

## Abstract

Breastfeeding provides optimal infant nutrition; however, <50% of infants are exclusively breastfed (EBF) for 6 months. We aimed to describe breastfeeding practices and their effects on growth and mortality among a high-risk mother-infant cohort in rural Sierra Leone. This was a secondary analysis of data from a randomized nutrition intervention trial among undernourished pregnant women. The study’s primary outcomes were infant weight and length gains at 6 weeks of age. We included 1270 singleton infants in the analysis, with 1092 (85.6%) having 24-week outcome data. At 6 weeks, 88% were EBF, but the rate of EBF decreased to 17% at 24 weeks. The EBF infants at 6 weeks had improved length (difference of 0.9 mm/week; 95% CI 0.4 to 1.3; *p* < 0.001) and weight (difference of 40 g/week; 95% CI 24 to 53; *p* < 0.001) gains compared to the non-EBF infants. At 12 weeks, the EBF infants had improved weight (difference of 12 g/week; 95% CI 2 to 22; *p* = 0.024) gain. The EBF infants had lower mortality than the infants who were not EBF (hazard ratio of 0.39; 95% CI 0.18 to 0.84; *p* = 0.017). In summary, the infants who were EBF had greater weight and length gain and reduced mortality than those who were not EBF. Efforts to improve breastfeeding should thus be prioritized to improve infant health.

## 1. Introduction

Breast milk is the ideal food for young infants, and breastfeeding one of the most effective ways to ensure a child’s health and survival [1,2,3,4]. Breast milk is safe, always at the appropriate temperature, ready to feed, available even in environments with poor sanitation and unsafe drinking water, and contains antibodies to protect against many common childhood diseases [2,5]. In light of this, the World Health Organization (WHO) and the United Nations Children’s Fund recommend exclusive breastfeeding for all children for the first six months of life, including in the setting of maternal HIV infections with antiretroviral therapy [6,7]. In addition to the benefits to the infant, breastfeeding is also advantageous for the mother, as it protects against postpartum hemorrhage, may allow for improved birth spacing, and reduces the risk of breast and ovarian cancers [8]. Optimal infant feeding includes exclusive breastfeeding (EBF) for six months and continued breastfeeding for up to 2 years, with the introduction of complementary foods beginning at 6 months [9]. Notably, even small tastings of liquids and foods other than breastmilk are associated with a shortened duration of EBF [10].

Despite the above-mentioned benefits of EBF and the many efforts, guidelines and policies to improve breastfeeding practices globally, only 44% of infants are breastfed exclusively from birth through 6 months of life [11]. Although several countries have made progress toward improving EBF practices, many low- and middle-income countries lag behind [12]. The reported prevalences of EBF from 2000 to 2017 were 33% in West, 53% in East, 24% in Central, and 57% in Southern Africa [13,14]. These data demonstrate that in many West and Central African countries, the prevalence of EBF is below the WHO and UNICEF recommendation of 50% [15]. Nearly all newborns (98%) in Sierra Leone initiate breastfeeding within an hour of birth [16], and 54% are exclusively breastfed; however, a recent study in Pujehun District found that only three-quarters of the infants continued with EBF after one month and only one-third of the infants were exclusively breastfed when they were 4–5 months old [17].

Due to the seemingly paradoxical situation of the low adherence to EBF in rural Sierra Leone despite the many known benefits of breastfeeding, we aimed to evaluate EBF practices among Sierra Leonean mother-infant pairs among a cohort of mothers who were undernourished during pregnancy. Specifically, we aimed to (i) determine the duration of EBF, (ii) examine the associations between the duration of EBF and infant anthropometry, and (iii) examine if EBF impacted the infant mortality rate among infants in rural Sierra Leone. To this end, we performed a secondary analysis of infant data from a randomized nutrition intervention trial for pregnant women with undernutrition.

## 2. Materials and Methods

### 2.1. Study Design

This was a secondary analysis of infant clinical outcomes from a randomized, controlled trial in which pregnant women with undernutrition in rural Sierra Leone were randomized to a bundle of specialized supplementary food and anti-infective interventions or randomized to the standard of care [18]. Pregnant women experiencing undernutrition, identified by a mid-upper arm circumference (MUAC) of ≤23 cm and a fundal height of <35 cm as an estimate for gestational age, were recruited from 43 government antenatal clinics located in the Pujehun and Western Rural Area Districts of Sierra Leone [18,19]. The exclusion criteria included gestational diabetes, hypertension, and severe anaemia.

### 2.2. Study Interventions

The primary trial included a bundled intervention of a daily ration of a locally formulated, ready-to-use supplementary food (RUSF) and antimicrobial medications. The RUSF, locally named Mama Dutasi, contained skimmed milk powder, pearl millet, peanut paste, sugar, whey protein isolate and vegetable oil. It provided 520 kcal, 18 g of excellent protein and 100% of the RDA for most of the micronutrients required during pregnancy [19]. The RUSF was provided with two doses of 1 g of azithromycin, an intermittent preventative treatment for malaria, with 1500 mg/75 mg of sulfadoxine-pyrimethamine (SP) monthly starting in the second trimester, combined with testing and treatment with metronidazole for bacterial vaginosis and a dose of albendazole for deworming. Those women randomized to the standard of care received a daily package of 250 g of corn–soy blended flour (SuperCereal), 25 g of palmolein oil, 60 mg of iron and 400 µg of folic acid, along with one dose of albendazole and at least three doses of SP starting in the second trimester. The mothers were followed every 2 weeks for anthropometry assessments, study food rations and medications until they delivered. No intervention was provided thereafter. Full details of the design of the parent trial, procedures and intervention have been published [18,19].

### 2.3. Study Assessments

The infants were examined and measured within 72 h of birth and at 6 weeks, 12 weeks and 24 weeks of age. At each visit, infant weight, length, head circumference, MUAC, morbidity, feeding practices and survival were assessed by study staff. Study staff obtained nude weights accurate to 5 g using a digital scale (Seca 334; Hamburg, Germany), recumbent length to 0.1 cm measured in triplicate with a rigid height board (Seca 417; Hamburg, Germany) and head circumference and left arm MUAC using an insertion tape accurate to 0.1 cm. Since newborns experience weight loss of up to 10% in the first week of life, prompt assessments were needed to maintain data accuracy. A trained, dedicated birth measurement team was created to travel to the newborns and collect these measurements as soon as possible. Mothers or clinical birth attendants placed a call to the team at the time of delivery. This resulted in 45% of measurements being taken within 24 h and 79% of measurements being taken within 72 h.

At each follow-up visit, the mothers were interviewed about breastfeeding practices. If they reported breastfeeding, they were asked if the infant had received any other liquids or foods in addition to breast milk. These foods were categorized as water, a fluid containing milk (e.g., formula), native herbs, glucose water and solid foods (e.g., cereal porridge). Native herbs referred to mostly seed-bearing and aromatic plants or other shrubs, vines and trees that are used as herbal remedies and grow naturally in Sierra Leone. Glucose water was defined as a commercially prepared substance that was mostly water with a small amount of sugar added. Participants that missed three consecutive visits were considered lost to follow-up. Study staff attempted home visits for any patient lost to follow-up. For infants identified as deceased, the date of death was recorded.

### 2.4. Statistical Analyses

Data were collected using clinic management cards and then double-entered into an electronic Microsoft Access database. Databases were compared for discrepancies, and all discrepancies were resolved after examining the original data card. After confirming the data, the database was de-identified and locked for analysis. Survey responses for infant feeding practices from follow-up visits were reviewed and categorized as (1) exclusive breastfeeding (including medications and oral rehydration solution), (2) additional milk products, (3) additional water, (4) additional glucose water and (5) solid foods. An infant was considered exclusively breastfed at a visit if a mother reported providing no other food.

All singleton live births with at least two post-natal follow-up visits were included in the current analysis. Descriptive statistics were used to characterize the study population. Changes in infant weight and length were calculated as grams/week or millimeters/week. We used absolute values and not z-scores as all infant visits occurred at standard times, i.e., 6 weeks, 12 weeks and 24 weeks. To estimate differences in the rates of weight and length gains between each time point by breastfeeding status assessed at each time point, we performed a generalized linear regression with adjustment for baseline anthropometrics (weight or length, respectively), maternal intervention and infant sex. For example, to compare rates of weight gain between birth and week 6 by breastfeeding status, rates of weight gain were computed and breastfeeding status as documented at week 6 (EBF vs. not) was the comparison of interest. Our data were not able to distinguish between individuals who stopped EBF at week 2 and those who stopped week 4, for example. The regression adjustments were chosen a priori based on their expected predictive ability. The normality of residuals and homoscedasticity were assessed and deemed not violated prior to model implementation. From these regressions, 95% confidence intervals were derived, and *p*-values were estimated.

After confirming proportional hazards via inspection of log-minus-log plots, time-to-event for mortality stratified by EBF status at week 6 was analyzed using a Cox proportional hazards regression adjusted for maternal intervention, maternal age and infant sex. Maternal age was included based on its predictive ability for mortality in this population [20]. Defaulters were censored at the time of the first missed visit.

## 3. Results

### 3.1. Characteristics of the Study Participants

**Figure 1:**
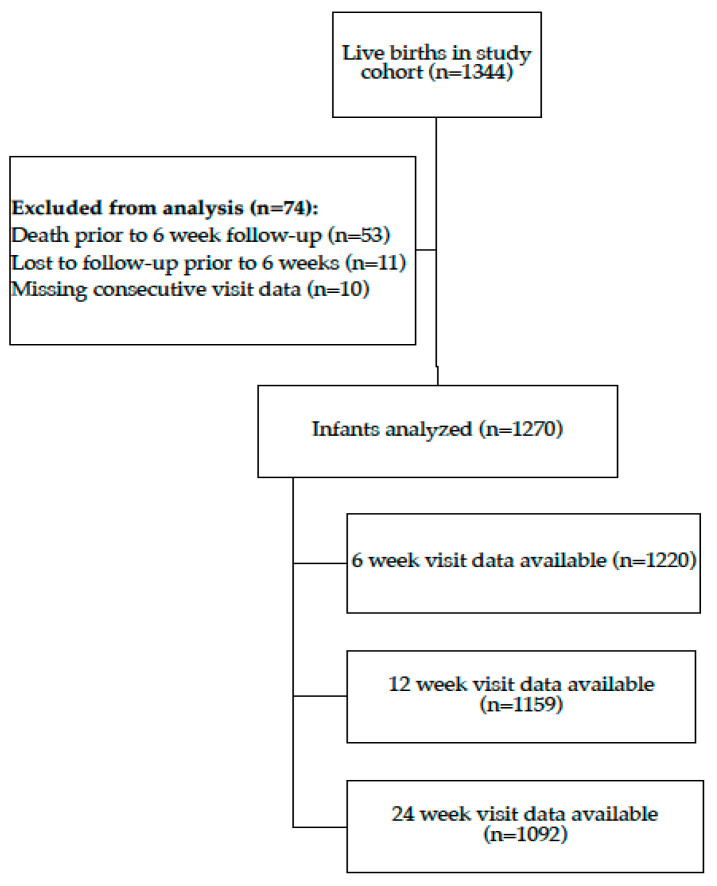
Flow chart of the participant inclusion process. Live-born infants of mothers enrolled in the primary clinical trial with at least two follow-up visits were included in this secondary study.

Among the 1344 singleton live births analyzed in the primary trial report, 1270 were included in this analysis (Figure 1, Table 1). Of these 1270 infants, 1092 had data available at the 24-week follow-up visit.

The mothers of the infants were young, with a median age of 20 (Q1 18, Q3 24) years, and for 522 (41.1%), this was their first child (Table 1). Most mothers had primary or no schooling (684, 54%). The mothers lived in rural settings, with a minority having electricity in their homes (106, 8.3%). Most households had clean water sources (1125, 88.5%) and improved toileting facilities (797, 62.7%).

### 3.2. Outcomes of the Rates of Exclusive Breastfeeding

At 6 weeks of life, nearly 90% of the infants were exclusively breastfed (Table 2). The infants who were not exclusively breastfeeding were mainly receiving water in addition to breastmilk. The rate of EBF decreased at each subsequent visit, with only approximately 17% of infants continuing EBF at the 24-week visit and most receiving complementary foods (Table 2).

### 3.3. Effects of Exclusive Breastfeeding on Infant Growth and Mortality

**Figure 2:**
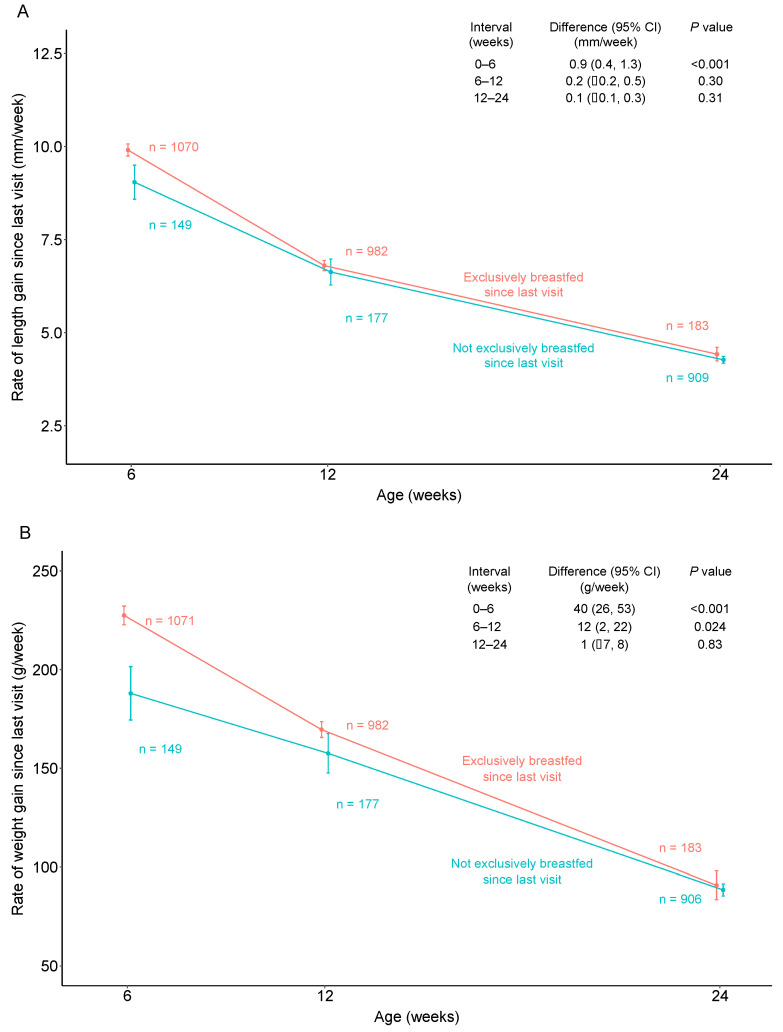
Comparison of the rates of length gain (**A**) and weight gain (**B**) by exclusive breastfeeding status from 6 to 24 weeks of age. Changes in infant weights and lengths were calculated as grams/week and millimeters/week, respectively. We performed a generalized linear regression with adjustments for baseline anthropometrics, maternal intervention and infant sex. The regression adjustments were chosen a priori on the basis of their expected predictive ability. The normality of the residuals and homoscedasticity were assessed and deemed not violated prior to model implementation. From these regressions, 95% confidence intervals were derivd and *p*-values were estimated.

The infants that were exclusively breastfed at the 6-week visit had improved lengths (difference of 0.9 mm/week; 95% CI 0.4 to 1.3; *p* < 0.001) and weights (difference of 40 g/week; 95% CI 26 to 53; *p* < 0.001) compared with the infants that were not exclusively breastfed (Figure 2). At the 12-week visit, the benefit of EBF on weight gain persisted (difference of 12 g/week; 95% CI 2 to 22; *p* = 0.024), though the benefit to length gains had waned (Figure 2). By 24 weeks, there were no significant differences in weight gain or length gain between the infants that were exclusively breastfed and those that were not.

**Figure 3:**
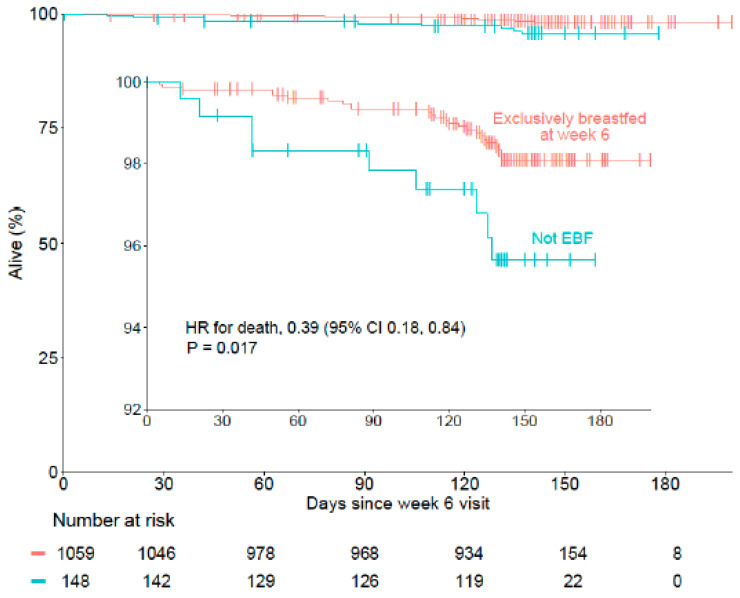
Time-to-event for mortality stratified by EBF status at week 6 was analyzed using a Cox proportional hazards regression adjusted for maternal intervention, maternal age and infant sex. The defaulters were censored at the time of the first missed visit. Displayed is a larger, full-scale figure with an inset limited scale figure to provide better detail of the curves. Abbreviations: CI, confidence interval; EBF, exclusively breastfed.

Among the cohort analyzed, there were 37 (2.9%) deaths by the 24-week follow-up period. The infants who were exclusively breastfed at week 6 had a lower mortality hazard than the infants who were not exclusively breastfed at week 6 (Cox proportional hazard ratio of 0.39; 95% CI 0.18 to 0.84; *p* = 0.017) (Figure 3).

## 4. Discussion

Breastfeeding encourages healthy infant growth and protects young infants from many serious infectious diseases; however, current exclusive breastfeeding rates remain below international goals. Improving breastfeeding rates globally could prevent over 800,000 deaths in children under five years of age annually [8]. Policymakers and governments should continue to engage on this topic as a lack of exclusive breastfeeding is associated with developmental delays and economic losses totaling over USD 300 billion or 0.5% of the world’s gross income [8]. Encouragingly, current trends in exclusive breastfeeding prevalence are improving in sub-Saharan Africa [21].

Here, we report a rate of EBF of >80% among rural mothers, which is higher than previously reported rates of EBF in Sierra Leone [16,17]. The 2021 Sierra Leone National Nutritional Survey reported a high EBF rate compared to many other settings, with early initiation at 89%, exclusivity at 53% and the proportion of children continuing to breastfeed to 23 months at 98% [16]. Although we report the encouraging finding of a higher proportion of exclusive breastfeeding among our cohort, we cannot ascertain why the EBF rate was higher among our cohort. This high rate of EBF may have resulted from caregivers having frequent access to healthcare providers at the health facilities and in the community who were educated on the importance of EBF [22]. The mothers and their infants in our cohort received no specific intervention targeted at improving EBF practices but only received one-on-one counseling while meeting with study nurses. It is plausible that feeding practices in the studied communities may have been influenced by the presence of long-term nutrition-specific projects implemented by the study team in the district. Additionally, these results may have been influenced by the innovative interventions towards increasing breastfeeding nationwide in Sierra Leone. A survey performed in Freetown, Sierra Leone, demonstrated that nutrition education and sensitization provided to women at the facility level improved the rate of early initiation and exclusive breastfeeding practices, supporting this hypothesis [23]. Furthermore, a recent systematic review of infants and young children feeding practices in sub-Saharan Africa reported a 102% increase in exclusive breastfeeding at 3 months with the implementation of breastfeeding educational interventions [24].

We also demonstrated that infants who were exclusively breastfed at 6 weeks and 12 weeks of life had higher rates of weight gain when compared to infants who were not exclusively breastfed. Similarly, the infants who were exclusively breastfed had better length gains at 6 weeks of life, with a trend toward a positive effect at 12 weeks. Previous studies investigating EBF among infants in the Gambia region reported similar benefits regarding weight gain but did not report an effect on length among their cohort [25]. This was in contrast to a cohort in Malawi that did not find a significant weight or length benefit among exclusively breastfed infants 0–6 months old [26]. Nevertheless, frequent growth faltering continues to be an issue among populations in low- and middle-income countries (LMICs) with high EBF rates [25,27]. The benefit of EBF on infant weight and length gains had waned by the 24-week visit in our cohort. This was not surprising as this represents the time when complementary feeding should be introduced to provide additional nutritional support to encourage adequate infant growth and development. However, longitudinal studies have demonstrated that exclusive breastfeeding for 6 months is associated with greater heights and fat-free masses up to 5 years of age, suggesting that improvements in linear growth may be sustained among exclusively breastfed infants [28].

Most importantly, we demonstrated that mortality risk was reduced among infants who were exclusively breastfed compared to infants who were not exclusively breastfed at 6 weeks of life. This finding has been reported in other settings, and a 1% increase in the number of exclusively breastfed infants at 6 months has been estimated to decrease the infant mortality rate by 0.5 per 100 infants [21]. While this was an important finding, we were unable to ascertain the etiology of this benefit in our dataset. It is possible that those who were exclusively breastfed had lower rates of infectious diseases such as diarrhea and pneumonia compared to the infants that were not exclusively breastfed, thereby reducing the risk of infant morbidity and mortality [1,2,4]. EBF to 6 months has been reported to decrease the risk of diarrheal diseases by up to 24% [24]. An alternative explanation is that the mothers may have discontinued EBF if they perceived their infants as “sick”; thus, mortality was increased due to factors other than EBF practices.

It is important to recognize that bias may have been introduced by asking the mothers about their breastfeeding practices at each visit. We were also unable to identify the reasons for non-exclusive breastfeeding in this study. Mothers discontinue EBF prior to six months for various reasons, including the perceptions about breastmilk alone being unable to meet their infants’ nutritional requirements, shorter maternity leaves, and sociocultural pressures to provide their child with water and other foods [17,29]. Previous studies in Sierra Leone and, specifically, in Pujehun District have identified that the major reasons for the interruption of exclusive breastfeeding were the maternal perceptions that their babies were hungry, that they felt their milk supplies were insufficient or their infants crying [17]. Additionally, the mothers reportedly provided water and/or native herbs for “bad stools.” [17]. A large systematic review of data from LMICs had consistently found that maternal perceptions about insufficient supply and socio-cultural factors related to infant nutrition were common reasons for the discontinuation of EBF [29]. Given these identified factors, additional efforts should be made to improve maternal and societal education around the benefits of EBF and the risks of introducing additional substances to young infants.

The strengths of our study included its large study population, standardized collection of all anthropometric measurements by specially trained study staff, robust follow-up growth and mortality data collection, and longitudinal presentation of breastfeeding data amongst a vulnerable and understudied group, i.e., undernourished women. However, this study also had multiple limitations. First, the comparison of EBF vs. non-EBF was observational in nature and thus was subject to confounding. This limited inference to the level of association rather than causality. Second, this was a post hoc analysis for which the original study was not powered, and thus, the *p*-values may have inflated type I errors. Third, the study population comprised infants of pregnant women suffering from undernutrition, which may not have reflected the general population of infants. This was particularly relevant to the extent that breastmilk production and quality are impacted by maternal undernutrition and these factors may influence breastfeeding practices. Fourth, a majority of the mothers in this study ceased EBF between 12 and 24 weeks, but our data lacked the granularity needed to probe the possible associations with that transition in this majority of mothers. Fifth, we did not collect data on the amount of non-EBF feeding, and it is possible that the amount impacted the outcomes. Finally, our findings are applicable to rural Sierra Leone and may not apply to other settings.

## 5. Conclusions

We found high rates of exclusive breastfeeding among a mother-infant cohort in rural Sierra Leone. Exclusive breastfeeding was associated with greater weight and length gains at 6 weeks of life and a reduced risk of mortality. Governments should prioritize evidence-based interventions to improve EBF rates and optimize child potential since efforts to improve EBF decrease infant morbidity and mortality.

## Figures and Tables

**Table 1:** Baseline maternal characteristics.

Characteristic ^1^	Mother N = 1270
Median (Q1, Q3) age of mother (years)	20 (18, 24)
Education level of mother	
None, *n* (%)	408 (32.1)
Primary school, *n* (%)	276 (21.7)
Secondary school or greater, *n* (%)	586 (46.1)
Home with a thatch or plastic roof, *n* (%)	196 (15.4)
Home with a radio, *n* (%)	872 (68.7)
Home with a bicycle, *n* (%)	277 (21.8)
Home with electricity, *n* (%)	106 (8.3)
Clean water source, *n* (%)	1125 (88.5)
Animals sleep in the home, *n* (%)	755 (59.4)
Improved toilet ^2^, *n* (%)	797 (62.7)
Number of previous pregnancies, *n* (%)	
None	522 (41.1)
One	328 (25.8)
More than one	420 (33.1)

Abbreviations: Q1, lower quartile; Q3, upper quartile. ^1^, values expressed as means (SDs) unless otherwise indicated; ^2^, includes private flush plumbing and locally improved pit in contrast to open pit, stream, bush and other non-improved facilities.

**Table 2:** Feeding practices of the studied infants.

Infant Feeding	Infant Age		
	6 Weeks N = 1220	12 Weeks N = 1159	24 Weeks N = 1092
Exclusively breastfed, *n* (%)	1070 (87.7%)	982 (84.7%)	183 (16.8%)
Water alone, *n* (%)	80 (6.6%)	104 (9.0%)	143 (13.1%)
Glucose water ^1^, *n* (%)	13 (1.1%)	6 (0.5%)	7 (0.6%)
Milk product (formula) ^2^, *n* (%)	22 (1.8%)	23 (2.0%)	31 (2.8%)
Native herbs ^3^, *n* (%)	29 (2.4%)	15 (1.3%)	9 (0.8%)
Solids ^4^, *n* (%)	5 (0.4%)	29 (2.5%)	719 (65.8%)

^1^, commercially prepared substance that is mostly water with a small amount of sugar added; ^2^, foods or beverages made from the milk of animals, usually cows, goats or sheep; ^3^, refers to seed-bearing, aromatic or useful shrubs, vines and trees that grow naturally in Sierra Leone; ^4^, includes infant cereals, proteins, fruits, vegetables, grains and more.

## Data Availability

The data presented in this study are available on request from the corresponding author. The data are not publicly available due to the presence of possible participant identifiable content after de-identification.

## References

[1] Khan J., Vesel L., Bahl R., Martines J.C. (2015). Timing of breastfeeding initiation and exclusivity of breastfeeding during the first month of life: Effects on neonatal mortality and morbidity—A systematic review and meta-analysis. Matern. Child Health J..

[2] Lamberti L.M., Zakarija-Grković I., Fischer Walker C.L., Theodoratou E., Nair H., Campbell H., Black R.E. (2013). Breastfeeding for reducing the risk of pneumonia morbidity and mortality in children under two: A systematic literature review and meta-analysis. BMC Public Health.

[3] Horta B.L., Loret de Mola C., Victora C.G. (2015). Breastfeeding and intelligence: A systematic review and meta-analysis. Acta Paediatr..

[4] Lamberti L.M., Fischer Walker C.L., Noiman A., Victora C., Black R.E. (2011). Breastfeeding and the risk for diarrhea morbidity and mortality. BMC Public. Health.

[5] Smith E.R., Hurt L., Chowdhury R., Sinha B., Fawzi W., Edmond K.M., Group N.S. (2017). Delayed breastfeeding initiation and infant survival: A systematic review and meta-analysis. PLoS ONE.

[6] World Health Organization (2002). Global Strategy for Infant and Young Child Feeding.

[7] World Health Organization, United Nations Children’s Fund (2016). Guideline: Updates on HIV and Infant Feeding: The Duration of Breastfeeding, and Support from Health Services to Improve Feeding Practices among Mothers Living with HIV.

[8] Rollins N.C., Bhandari N., Hajeebhoy N., Horton S., Lutter C.K., Martines J.C., Piwoz E.G., Richter L.M., Victora C.G., Group L.B.S. (2016). Why invest, and what it will take to improve breastfeeding practices?. Lancet.

[9] Sankar M.J., Sinha B., Chowdhury R., Bhandari N., Taneja S., Martines J., Bahl R. (2015). Optimal breastfeeding practices and infant and child mortality: A systematic review and meta-analysis. Acta Paediatr..

[10] Stern J., Funkquist E.L., Grandahl M. (2023). The association between early introduction of tiny tastings of solid foods and duration of breastfeeding. Int. Breastfeed. J..

[11] Zong X., Wu H., Zhao M., Magnussen C.G., Xi B. (2021). Global prevalence of WHO infant feeding practices in 57 LMICs in 2010-2018 and time trends since 2000 for 44 LMICs. EClinicalMedicine.

[12] Perez-Escamilla R., Tomori C., Hernandez-Cordero S., Baker P., Barros A.J.D., Begin F., Chapman D.J., Grummer-Strawn L.M., McCoy D., Menon P. (2023). Breastfeeding: Crucially important, but increasingly challenged in a market-driven world. Lancet.

[13] Victora C.G., Bahl R., Barros A.J., França G.V., Horton S., Krasevec J., Murch S., Sankar M.J., Walker N., Rollins N.C. (2016). Breastfeeding in the 21st century: Epidemiology, mechanisms, and lifelong effect. Lancet.

[14] Bhattacharjee N.V., Schaeffer L.E., Marczak L.B., Ross J.M., Swartz S.J., Albright J., Gardner W.M., Shields C., Sligar A., Schipp M.F. (2019). Mapping exclusive breastfeeding in Africa between 2000 and 2017. Nat Med.

[15] World Health Organization, United Nations Children’s Emergency Fund (2014). Global Nutrition Targets 2025: Breastfeeding Policy Brief (WHO/NMH/NHD/14.7).

[16] Ministry of Health and Sanitation Sierra Leone and UNICEF Sierra Leone (2021). Sierra Leone National Nutrition Survey 2021.

[17] van Breevoort D., Tognon F., Beguin A., Ngegbai A.S., Putoto G., van den Broek A. (2021). Determinants of breastfeeding practice in Pujehun district, southern Sierra Leone: A mixed-method study. Int. Breastfeed. J..

[18] Hendrixson D.T., Smith K., Lasowski P., Callaghan-Gillespie M., Weber J., Papathakis P., Iversen P.O., Koroma A.S., Manary M.J. (2021). A novel intervention combining supplementary food and infection control measures to improve birth outcomes in undernourished pregnant women in Sierra Leone: A randomized, controlled clinical effectiveness trial. PLoS Med..

[19] Hendrixson D.T., Koroma A.S., Callaghan-Gillespie M., Weber J., Papathakis P., Manary M.J. (2018). Use of a novel supplementary food and measures to control inflammation in malnourished pregnant women in Sierra Leone to improve birth outcomes: Study protocol for a prospective, randomized, controlled clinical effectiveness trial. BMC Nutr..

[20] Koroma A.S., Ellie M., Bangura K., Iversen P.O., Hendrixson D.T., Stephenson K., Manary M.J. (2023). Supplementary feeding and infection control in pregnant adolescents-A secondary analysis of a randomized trial among malnourished women in Sierra Leone. Matern. Child. Nutr..

[21] Pretorius C.E., Asare H., Kruger H.S., Genuneit J., Siziba L.P., Ricci C. (2021). Exclusive Breastfeeding, Child Mortality, and Economic Cost in Sub-Saharan Africa. Pediatrics.

[22] Kinshella M.W., Prasad S., Hiwa T., Vidler M., Nyondo-Mipando A.L., Dube Q., Goldfarb D., Kawaza K. (2021). Barriers and facilitators for early and exclusive breastfeeding in health facilities in Sub-Saharan Africa: A systematic review. Glob. Health Res. Policy.

[23] Jalloh Y.K. (2012). Evaluation of Knowledge, Attitudes, and Practices of Mothers in Relation to Exclusive Breastfeeding and Complementary Feeding of Their Children in Clinics Served by Helen Keller International in Freetown, Sierra Leone after the Introduction of a Multi-Faceted Intervention. Master’s Thesis.

[24] Lassi Z.S., Rind F., Irfan O., Hadi R., Das J.K., Bhutta Z.A. (2020). Impact of Infant and Young Child Feeding (IYCF) Nutrition Interventions on Breastfeeding Practices, Growth and Mortality in Low- and Middle-Income Countries: Systematic Review. Nutrients.

[25] Eriksen K.G., Johnson W., Sonko B., Prentice A.M., Darboe M.K., Moore S.E. (2017). Following the World Health Organization’s Recommendation of Exclusive Breastfeeding to 6 Months of Age Does Not Impact the Growth of Rural Gambian Infants. J. Nutr..

[26] Kamudoni P., Maleta K., Shi Z., Holmboe-Ottesen G. (2015). Exclusive breastfeeding duration during the first 6 months of life is positively associated with length-for-age among infants 6-12 months old, in Mangochi district, Malawi. Eur. J. Clin. Nutr..

[27] Sithamparapillai K., Samaranayake D., Wickramasinghe V.P. (2022). Timing and pattern of growth faltering in children up-to 18 months of age and the associated feeding practices in an urban setting of Sri Lanka. BMC Pediatr..

[28] Heltbech M.S., Jensen C.L., Girma T., Abera M., Admassu B., Kaestel P., Wells J.C.K., Michaelsen K.F., Friis H., Andersen G.S. (2023). The Associations of Breastfeeding Status at 6 Months with Anthropometry, Body Composition, and Cardiometabolic Markers at 5 Years in the Ethiopian Infant Anthropometry and Body Composition Birth Cohort. Nutrients.

[29] Balogun O.O., Dagvadorj A., Anigo K.M., Ota E., Sasaki S. (2015). Factors influencing breastfeeding exclusivity during the first 6 months of life in developing countries: A quantitative and qualitative systematic review. Matern. Child. Nutr..

